# Impact of Various Atrial Fibrillation Treatment Strategies on Length of Stay in the Emergency Department and Early Complications—3 Years of a Single-Center Experience

**DOI:** 10.3390/jcm13010190

**Published:** 2023-12-29

**Authors:** Tomasz Kłosiewicz, Hanna Cholerzyńska, Wiktoria Antonina Zasada, Amira Shadi, Jakub Olszewski, Patryk Konieczka, Roland Podlewski, Mateusz Puślecki

**Affiliations:** 1 Department of Emergency Medicine, Poznan University of Medical Sciences, 7 Rokietnicka Street, 60-608 Poznań, Poland; 79054@student.ump.edu.pl (H.C.); 81576@student.ump.edu.pl (W.A.Z.); p.konieczka@ump.edu.pl (P.K.); rpodlew@ump.edu.pl (R.P.); mateusz.puslecki@ump.edu.pl (M.P.); 2 College of Emergency Physicians in Poland, 5 Truflowa Street, 62-070 Dopiewiec, Poland; a.shadi@plmr.med.pl (A.S.); j.olszewski@plmr.med.pl (J.O.)

**Keywords:** atrial fibrillation, electric countershock, emergency department, length of stay, rhythm control

## Abstract

Atrial fibrillation (AF) is the most common arrhythmia presenting in emergency departments (EDs), vastly increasing mainly due to society’s lifestyles leading to numerous comorbidities. Its management depends on many factors and is still not unified. Aims: The aim of this study was to compare different AF management strategies in the ED and to evaluate their influence on the length of stay (LOS) in the ED and their safety. We analyzed medical records over 3 years of data collection, including age, primary AF diagnosis, an attempt to restore sinus rhythm, complications, and length of stay. Patients were divided into three groups according to the treatment method received: only pharmacological cardioversion (MED), only electrical cardioversion (EC), and patients who received medications followed by electrical cardioversion (COMB). We included 599 individuals in the analysis with a median age of 71. The restoration of sinus rhythm and LOS were as follows: MED: 64.95%, 173 min; COMB: 87.91%, 295 min; SH: 92.40%, 180 min. The difference between the MED and EC strategies, as well as MED and COMB, was statistically significant (*p* < 0.001 in both). The total number of complications was 16, with a rate of 32.67%. The majority of them followed a drug administration, and the most common complication was bradycardia. Electrical cardioversion is a safe and effective treatment strategy in stable patients with AF in the ED. It is associated with a shortened LOS. Medication administration preceded the majority of complications.

## 1. Introduction

Atrial fibrillation (AF) is the most common cardiac arrhythmia encountered in the emergency department (ED), constituting a substantial proportion of total ED admissions, ranging from 3.3% to 10% and currently ranking as a primary cause for geriatric visits [1,2,3]. Its pervasive prevalence is on the ascent, a consequence attributed to societal lifestyle changes, which, in turn, contribute to an increase in associated diseases and symptoms [4,5]. Consequently, the number of acute ED admissions for AF is escalating. The exigency for effective AF management is underscored by its pivotal role in preventing heart failure, mitigating the risk of stroke, and alleviating associated symptoms.

In the acute phase, clinicians are confronted with three primary management options: pharmacological cardioversion, electrical cardioversion, or heart rate control. The selection among these options is based on multifaceted considerations related to the patient’s clinical presentation [6]. While the management of hemodynamically unstable patients may be characterized with relative clarity, the decision-making process for stable patients involves a nuanced choice between a pharmacological treatment strategy and electrical cardioversion. Factors that come into play in such situations include the patient’s condition, available resources, organizational constraints, and local practices.

The overarching goal remains consistent: to address acute symptoms, identify and rule out underlying causes, initiate anticoagulation, and facilitate discharge with subsequent outpatient follow-up. Recent studies demonstrate the safety of performing cardioversion in the ED without necessitating hospitalization, contingent upon the fulfillment of specific criteria [7]. This approach not only proves to be cost effective for hospitals, but also serves to curtail admissions to cardiology wards, consequently reducing the hospital stay duration and the associated expenses. As highlighted by Sacchetti et. al., the median hospitalization cost for ED cardioversion stands at USD 5460, a stark contrast to the USD 23,202 associated with hospitalization without rhythm control [8,9]. Given the substantial challenges posed by the workload and overcrowding prevalent in emergency departments, strategies that optimize ED functioning and enhance patient flow assume paramount importance. This study seeks to scrutinize and compare different cardioversion strategies performed by emergency physicians, aiming to evaluate their impact on the ED length of stay (LOS) and the occurrence of early complications.

## 2. Materials and Methods

### 2.1. Data Collection and Study Protocol

All the data used in this study were retrieved from the Hospital Information System used in our Emergency Department, filtered based on accordance with criteria established by the authors. The main inclusion criteria for this study were delineated as follows: (1) individuals aged above 18 years, (2) paroxysmal atrial fibrillation as the primary diagnosis at the time of presentation in the ED, (3) an attempted restoration of sinus rhythm, (4) the ED visit occurring between April 2019 and March 2022, (5) time onset of AF less than 24 h before presentation, unless patient was regularly taking anticoagulants. Conversely, exclusion criteria comprised spontaneous rhythm conversion, non-therapeutic international normalized ratio (INR) in patients taking vitamin K antagonists (VKAs), absence of regular intake of non-vitamin K antagonists or oral anticoagulants (NOACs) regularly within the last 4 weeks in patients with a history of chronic VKA or NOAC treatment, symptoms indicative of shock, absence of consent for the proposed treatment, hypokalemia (defined as serum potassium ion concentration less than 3.5 mmol/L), hyperthyroidism or hypothyroidism (defined as serum thyroid-stimulating hormone concentration lower than 0.27 mIU/L and higher than 4.20 mIU/L, respectively).

The collected data included age and sex as standard demographic indicators, as well as the 10th Revision of the International Classification of Diseases (ICD-10) diagnosis, the timing of the ED visit including time of admission to the ED, and the total length of stay in the ED. Additionally, data have also been sought on the use of antiarrhythmic agents, procedural sedation and analgesia (PSA), and synchronized cardioversion. The endpoint of treatment was defined as a success if sinus rhythm was restored and deemed a failure if this was not achieved, with the patient’s final disposition categorized either as discharge or admission. All the data were exported to spreadsheets, subjected to filtering, and scrutinized for potential discrepancies. Complications were defined as events occurring post-cardioversion that exerted an influence on the further treatment of the patient, thereby resulting in additional procedures or prolonged observation.

Patients were categorized into three distinct groups according to the treatment provided. Group 1 (medications only—MED) constituted of individuals exclusively administered antiarrhythmic drugs, such as amiodarone, propafenone, and antazoline, all available in our ED. Group 2 (shock only—EC) comprised patients subjected to initial electrical cardioversion as the primary intervention. Group 3 (combined—COMB) involved patients receiving medication initially, with subsequent electrical cardioversion in cases where the initial treatment did not yield success. The timing for assessing drug efficacy was unregulated and varied depending on the drug used, typically ranging from 1 h (phenazoline) to 6–8 h (amiodarone). The specific time was not consistently documented in the analyzed medical records, as physicians customarily recorded medical events retrospectively.

The choice of the cardioversion method was contingent upon physician expertise, evaluation of patient history, and assessment of indications and contraindications individually in every case. Factors considered included: atrial fibrillation duration, prior unsuccessful attempts at pharmacological cardioversion, and the availability of experienced personnel for sedation and electrical cardioversion. Notably, there were no pre-established departmental rules that dictated the therapeutic approach, and the current guidelines do not indicate the superiority of one method of cardioversion over another. Several considerations guided the selection of the drug for cardioversion, with the options limited to three pharmaceuticals available in our department: amiodarone, propafenone, and phenazoline. In instances where uncertainties arose, there was an option to seek guidance from the consulting physician—an emergency medicine specialist—providing an additional layer of expertise to inform decision making. This approach underscores the individualized nature of the decision-making process, recognizing that the selection of cardioversion methods and drugs necessitates a case-by-case assessment, with a reliance on clinician expertise, patient-specific factors, and the available resources within the department.

### 2.2. Safety Conditions for Sedation

In our department, the procedural sedation and analgesia (PSA) is performed according to “Unscheduled Procedural Sedation: A Multidisciplinary Consensus Practice Guideline” [10]. The minimum staffing requirements encompass two emergency medicine physicians and one or two paramedics or nurses. The patient undergoing PSA is located in the intensive care area where a singular medical practitioner is committed to the execution of the PSA procedure, obviating the necessity for an auxiliary anesthetist operating extraneous to the department. Comprehensive monitoring is available. Preceding the procedure, equipment is prepared for airway clearance and security, performing ventilation and resuscitation. Fasting time is regulated by the guidelines set forth in “An international multidisciplinary consensus statement on fasting before procedural sedation in adults and children” [11]. After PSA, the patient is observed by a physician until consciousness is regained, and is subsequently monitored and observed by a dedicated paramedic/nursing staff. The observation time after sedation, while not rigidly regulated, is adjusted according to the patient’s overall general condition. A patient who has fully regained consciousness and demonstrates the ability to walk and drink without vomiting may be safely discharged with the accompaniment of another responsible adult.

### 2.3. Outcomes

Outcomes in this study were defined as follows: (1) length of stay (LOS)—the time between triage and final decision regarding admission or discharge; (2) restoring sinus rhythm; (3) occurrence of any cardioversion-related complications, as previously defined.

### 2.4. Statistical Analysis

Statistical analysis was performed using the Statistica 12 software (Tibco Inc., Tulsa, OK, USA). Descriptive statistics of measurable variables were generated. Categorical variables were expressed as *n* [%]. Quantitative data were first checked for normal distribution with Shapiro–Wilk test. As they did not conform to the normal distribution, data were presented as median [interquartile range].

In order to elucidate distinctions among three distinct cohorts, an one-way analysis of variance (ANOVA) was conducted. Subsequently, given the inherent dissimilarities in group sizes, the Tukey test was selected as the suitable post hoc examination for further scrutiny. However, if heterogeneous variations were observed, a Games–Howell test was used, as appropriate. A *p*-value < 0.05 indicated statistical significance.

### 2.5. Ethical Considerations

This study adheres to the ethical guidelines outlined in opinion number KB 700/22 from the Poznan University of Medical Sciences’ Ethics Committee. According to the committee’s assessment, the nature of the research does not meet the criteria of a medical experiment, and therefore, formal consent is not deemed necessary. The study design and procedures were conducted following the approved ethical standards, ensuring the protection of participants’ rights and privacy.

## 3. Results

### 3.1. Study Population

**Figure 1:**
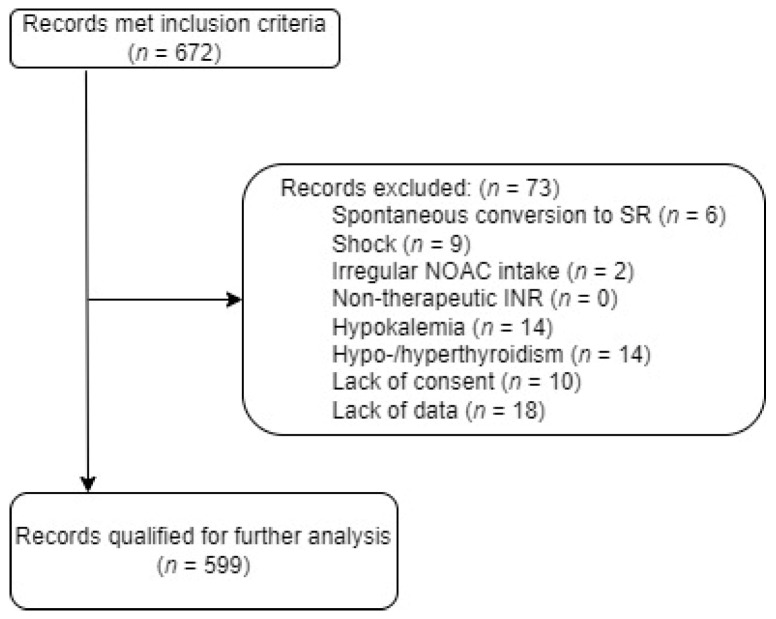
Study flowchart. SR—sinus rhythm, NOAC—novel oral anticoagulants, INR—international normalized ratio.

A comprehensive cohort of 672 individuals initially met the specified inclusion criteria. However, upon examination, 73 individuals were subsequently excluded, a visual representation of which is provided in Figure 1. The remaining 599 individuals were qualified for further in-depth analysis. The study flowchart illustrating the screening process is presented in Figure 1 for clarity and transparency. The median age of the cohort was determined to be 71 [62–79] years old. Remarkably, the age spectrum spanned from a minimum of 25 years to a maximum of 97 years, underscoring the diverse demographic composition of the study group. The main characteristics of the study group are presented in Table 1.

Antiarrhythmic agents constituted the exclusive treatment for AF in 61.93% of patients (*n* = 371), synchronized shock alone was applied in 13.198% of patients (*n* = 79), and a combined approach involving medications followed by synchronized shock was employed in 24.88% of patients (*n* = 149).

### 3.2. Effectiveness

Rhythm conversion was attained in 64.95% (*n* = 241) of patients in the MEDS group, 87.91% (*n* = 131) of patients in the COMB group, and 92.40% (*n* = 73) of patients in the EC group. Statistically significant differences were observed between the effectiveness of the MED and EC strategies (*p* < 0.001) and between the MED and COMB strategies (*p* < 0.001), while the difference between the COMB and EC strategies was not significant (*p* = 0.2549). Detailed information about the effectiveness of different drugs is presented in Table 2.

### 3.3. Length of Stay

The median length of stay for patients in the EC group was determined to be 180 [131–295] min. In comparison, patients in the MED group exhibited a median LOS of 173 [1120–275] min. Notably, the observed difference in LOS between the EC and MED groups was found to be statistically non-significant (*p* = 0.4125). The median LOS for patients receiving the combined treatment was determined to be 295 [214–392]. Significantly divergent LOS values were evident between the COMB group and both the EC group (*p* < 0.001) and the MED group (*p* < 0.001). These findings highlight a notable 1.5-fold reduction in LOS when comparing the EC strategy with the combined (COMB) strategy, underscoring the potential impact of treatment modalities on the duration of patient stay in the emergency department.

### 3.4. Complications

The total number of complications documented in this study was 16 (11 observed in male patients and 5 in female patients). Based on this number, the calculated complication rate for the whole group was determined to be 2.67%.

Noteworthy is the observation that medication administration preceded a majority of complications, with a total of nine incidents. Of these, the administration of antazoline amiodarone, and propafenone contributed to three complications. An additional four incidents occurred following procedural sedation analgesia, and three were a consequence of synchronized shock.

Among the complications, bradycardia emerged as the most frequently encountered, manifesting in seven patients. Other documented complications included: transient respiratory depression requiring temporary ventilation (four patients), hypotension (three patients), and chest pain without evidence of ischemia (one patient). Notably, one incident of cardiac arrest was observed, and this event was preceded by electrical cardioversion. There were no fatal complications. For a comprehensive overview of all incidents, including their frequency and nature, Supplementary Table S1 is provided. Additionally, Table 3 provides key information regarding various cardioversion strategies chosen by physicians.

## 4. Discussion

The management of atrial fibrillation in the emergency room demands a comprehensive approach, necessitating the integration of the physician’s extensive knowledge, a thorough patient history, careful consideration, and meticulous situation assessment. The rhythm control strategy aims to restore and maintain the patient’s sinus rhythm, employing either antiarrhythmic agents or electrical cardioversion [6]. Its application is particularly recommended to reduce AF-related symptoms and improve the quality of life in symptomatic patients with AF. The decision-making process regarding the choice of the most suitable strategy is intricate and multifaceted. It hinges on various factors, encompassing the patient’s individual characteristics and the accessibility of medications or procedures. Consequently, the responsibility for making this decision lies with the physician, as the overall effectiveness of both strategies appears comparable [12,13]. This underscores the nuanced nature of AF management, where tailored decisions are crafted based on individual patient profiles and the context of resource availability within the emergency room setting. Ultimately, the goal remains to optimize patient outcomes by addressing symptoms, improving quality of life, and navigating the intricate landscape of AF management with a judicious and patient-centric approach.

The restoration of sinus rhythm in hemodynamically stable patients in an ED is still a subject of discussion. Rogenstein et al. identified significant variability among ED physicians around the world [14]. Within Europe, pharmacological management is more common in Eastern Europe, and EC is more prevalent in Northern and Western Europe [12]. A recent EHRA-PATHS study, a project led by the European Society of Cardiology (ESC) and its constituent the European Heart Rhythm Association (EHRA), highlighted that interdisciplinary care for AF in Poland may be restrained by financial aspects [15].

Knudsen Pope et al. conducted an observational study, the findings of which revealed that direct current cardioversion was administered twice as frequently as pharmacological cardioversion. Interestingly, despite this disparity in utilization, no discernible differences in outcomes were observed between the two modalities. Notably, the study reported a significantly lower mortality rate associated with cardioversion, whether pharmacological or electrical, in comparison to the wait-and-see approach [16]. These results underscore the potential benefits of a proactive approach, whether through direct current cardioversion or pharmacological means, in mitigating adverse outcomes and mortality in the context of atrial fibrillation management. Stiell et al. undertook a comparative analysis, investigating the outcomes of two distinct approaches for cardioversion in atrial fibrillation. The study compared the pharmacological cardioversion followed by electrical cardioversion with exclusive electrical cardioversion. Notably, the findings of the analysis did not reveal any statistically significant differences in outcomes between those two strategies. The effectiveness of medication-only interventions was noted as 52% [17]. The reported rates in the literature range between 32% and 90%, depending on the administered drug [6]. However, electrical cardioversion demonstrated a remarkable success rate of 94.2% [18]. Notably, our findings align with the proposition that an exclusive electrical cardioversion approach proves more efficacious than both the medication-only and combined strategies.

Some authors have proposed that treatment with amiodarone for a duration of 1–6 weeks before receiving elective EC improves the restoration and maintenance of sinus rhythm. However, it is essential to note that the applicability of these results may not be extrapolated to emergency department (ED) settings unless patients are consistently treated with amiodarone on a daily basis pre-presentation [19]. The potential use of magnesium sulfate infusion in addition to the standard treatment of AF remains a subject of ongoing discussion, with multiple studies reporting its effectiveness despite its exclusion from the newest guidelines [20]. Furthermore, the intravenous administration of magnesium sulfate has been proposed to increase the likelihood of spontaneous conversion to sinus rhythm [21]. Its effectiveness in the prevention of AF and the lowering of the heart rate has been supported by the existing literature [22,23,24]. In our emergency department, magnesium was administered as an adjunctive treatment by physicians. The inherently subjective nature of this decision introduces the potential for variability in predicting the benefit of magnesium administration.

Interestingly, the comparison of the wait-and-see strategy and early cardioversion in patients with recent-onset symptomatic AF revealed comparable outcomes in achieving a return to sinus rhythm at 4 weeks [25]. However, Doyle and Reeves reported that in almost two thirds of patients in their study, AF spontaneously resolved before performing cardioversion [26]. The rate control approach in an ED appears to be beneficial, particularly when procedural sedation analgesia poses undue risks to general health conditions. However, it introduces the risk of readmission if there is no effect, contributing to the challenge of managing overcrowded EDs and necessitating organizational adjustments for planned ED readmissions [27].

The significant differences in LOS are noteworthy, with the shortest stays in the electrical cardioversion and medication-only groups. These findings, contrary to some literature, may be attributed to differing protocols, with some assuming a 6 h fasting period before PSA [28,29]. Current fasting and aspiration prevention recommendations advocate for tailoring fasting times to the presence of risk factors and the nature of ingested food or drink [11]. Moreover, fasting intervals are not absolute, and emergency physicians can often safely perform PSA promptly, eliminating the need for the additional wait time associated with a procedural organization, such as waiting for an anesthesiologist [30]. Notably, our study aligns with a US investigation reporting an 84% discharge rate within 4 h and a Canadian multicenter trial showing an LOS of 3.5 h, affirming our observations [31,32]. The potential wait time between a patient’s last meal and the initiation of PSA is an important component of performing electric cardioversion and a crucial factor that can influence LOS. Therefore, the comparison presented in this study underlines that there is no difference between performing pharmacological and electrical cardioversion regarding patients’ length of stay in the ED. A notable consideration is the absence of a specific time frame in the currently available guidelines regarding the recommended duration for patient observation after attempting to restore sinus rhythm. The ESC 2020 Guidelines for the Management of Atrial Fibrillation include information on the anticipated time for achieving sinus rhythm, which is commonly estimated at around 8 h [6]. However, the available literature indicates that 50% of the patients treated with pharmacological cardioversion who convert do so within 1 h, and 90% within 2 h [33]. The decision about the discharge is made by the physician, based on the patient’s status, the physician’s experience, and the current ED occupancy. Moreover, given the known association between the ED visit or longer length of stay and the risk of mental health disorders such as anxiety and depression, especially in the elderly population, extending the observation beyond 2 h seems inexpedient [34].

The complications observed in our study primarily resulted from the administration of medications, consistent with their well-known adverse effects. Similarly, Stiell et al. described the majority of adverse effects occurring after drug infusion [17]. However, the medication-only group exhibited the lowest adverse effect rate, which is a noteworthy finding considering the prevailing data indicating a higher incidence of adverse effects associated with drugs. Nonetheless, it is essential to note that the MEDS group was significantly larger. Following the administration of all drugs examined in our study (antazoline, propafenone, and amiodarone), the overall number of complications observed was equivalent. However, it is crucial to note that antazoline was employed more frequently than the other medications, leading to an uneven distribution in the incidence of complications. Moreover, it was the most successful drug in restoring the sinus rhythm during the observation period. Despite its efficacy and safety, it is noteworthy that the role of antazoline has yet to be incorporated into the guidelines of either the European Resuscitation Council or European Society of Cardiology [35]. Recently, a Polish study described antazoline as superior to propafenone and amiodarone in sinus rhythm conversion, with the adverse effect rate comparable and in line with our results. The authors recommended conducting randomized controlled trials to validate these findings [36]. While PSA was frequently associated with transient respiratory depression requiring temporary ventilation, this is a widely recognized adverse effect of this procedure and is not a limitation to its performance in the ED setting [10].

## 5. Limitations

Limitations are acknowledged in this study, with the foremost constraint being the reliance on data sourced exclusively from a single hospital. The length of stay in the ED depends on many factors. At each emergency department, the departmental organization of work, the number of physicians, and the composition of healthcare professionals may vary. The patient volume, the time required to organize electrical cardioversion procedures (including the necessary time period between a patient’s last meal and PSA initiation), and the wait time for laboratory results are also different. Consequently, caution must be exercised when generalizing the results of this study to other hospitals. In our opinion, however, the findings do demonstrate the dependencies on the treatment pathway. In the study, we did not assess factors such as arrhythmia recurrence, which, from the perspective of the workload of the ward, could also be relevant.

The retrospective nature of the study, relying on the analysis of medical records, introduces inherent limitations. Notable among these is the absence of certain data points that could have influenced procedural success, including the duration of symptoms, left atrium size, and details of prior procedures such as radiofrequency ablation. The selection of antiarrhythmic agents was beyond the researchers’ control, and while magnesium sulfate is not routinely recommended for atrial fibrillation, some physicians incorporate it based on studies suggesting its positive impact on restoring sinus rhythm. Physicians do not record in the documentation explicit reasons for the choice of a particular drug.

## 6. Conclusions

Based on our analysis, electrical cardioversion has shown to be an effective and safe treatment strategy for atrial fibrillation management in the emergency department, associated with a higher rate of successful sinus rhythm restoration. EC was as successful as a medication-only strategy in the context of achieving a shortened length of stay. Complications primarily occurred as a result of medication administration. These findings suggest that EC may be considered a viable approach in AF management in the ED, taking into account the potential risks associated with medication use. The implications of our study underscore the importance of further research and clinical studies to validate these conclusions, importantly, to refine optimal management strategies for AF in the ED setting. This ongoing exploration will contribute to enhancing the precision and efficacy of AF interventions in emergency healthcare settings.

## Figures and Tables

**Table 1:** The main characteristics of the study group.

Variable	Total	MED	EC	COMB	*p*-Value
Median age, years [IQR]	71 (62–79)	72 (63–81) §	68 (60–75)	67 (57–73) §	* < 0.05 § < 0.05
Median LOS, min [IQR]	206 (127–308)	173 (112–275) †	180 (131–295) §	295 (214–392) §,†	* < 0.05 § < 0.05 † < 0.05
Male, *n* (%)	291 (47.47)	162 (42.40) §,†	45 (56.96) §	84 (55.26) †	* 0.0082 § 0.0081 † 0.0093
Past medical history, *n* (%)					
Hypertension	209 (34.09)	148 (38.74) §	23 (29.11)	38 (25.00) §	* 0.0026 § 0.0201
Coronary artery disease	67 (10.51)	45 (11.78)	6 (7.59)	16 (10.52)	* 0.5428
Heart failure	24 (3.91)	15 (3.92)	2 (2.53)	7 (4.60)	* 0.7290
Diabetes mellitus	46 (7.50)	37 (9.68) §,†	2 (2.53) †	7 (4.60) §	* 0.0173 § 0.0222 † 0.0185
Chronic obstructive pulmonary disease	22 (3.59)	14 (3.66)	4 (5.06)	4 (2.36)	* 0.7476
History of hypo-/hyperthyroidism	39 (6.36)	22 (5.75)	6 (7.59)	11 (7.23)	* 0.7621

COMB—combined strategy, MED—medication-only strategy, EC—electrical cardioversion, * *p*-value for ANOVA analysis, §, †—*p*-values for post hoc test. For post hoc tests, only significant values were presented.

**Table 2:** Effectiveness of drugs in the MED group.

Drug	*n* (% of MED Group)	Effectiveness (%)
Amiodarone	133 (34.82)	40
Propafenone	14 (3.66)	83
Antazoline	155 (40.58)	88
Amiodarone, antazoline	64 (16.75)	53
Propafenone, antazoline	16 (4.19)	79

MED—medication-only strategy.

**Table 3:** Comparison of employed cardioversion strategies with the length of the patient’s stay (LOS), complication rate, and general effectiveness (defined as successful rhythm conversion).

Variable	Total	EC	MED	COMB	*p*-Value
LOS (min) ^1^	223 [144–328]	180 [131–295] †	173 [112–275] §	295 [214–392] §,†	* < 0.05 § < 0.05 † < 0.05
Effectiveness	74.29%	92.40% †	64.95% §,†	87.91% §	* < 0.05 § < 0.05 † < 0.05
Complication rate	2.67%	2.53%	0.81% §	7.38% §	* < 0.05 § < 0.05 † < 0.05

COMB—combined strategy, MED—medication-only strategy, EC—electrical cardioversion. * *p*-value for ANOVA analysis, §, †—*p*-values for post hoc test. For post hoc tests, only significant values were presented. ^1^ data are presented as median [IQR].

## Data Availability

The data presented in this study are available on request from the corresponding author.

## Supplementary Materials

The following supporting information can be downloaded at: https://www.mdpi.com/article/10.3390/jcm13010190/s1, Table S1: Complications of cardioversion and procedural sedation analgesia.
