# Supplementary material for: Impact of Various Atrial Fibrillation Treatment Strategies on Length of Stay in the Emergency Department and Early Complications—3 Years of a Single-Center Experience
Source: J Clin Med. 2023 Dec 29;13(1):190. doi: 10.3390/jcm13010190 (PMC10779744; doi:10.3390/jcm13010190)
Supplement: Supplementary file 1 [file jcm-13-00190-s001.zip › jcm-2713779-supplementary.pdf]

Supplementary Materials

Table S1. Complications of cardioversion and procedural sedation analgesia.

COMB - combined strategy, F - female, S.C. - synchronized cardioversion, Mg - Magnesium sulfate, M - male, MED - medications only strategy, SH - shock only strategy, TRD - transient respiratory depression requiring temporary ventilation

age	sex	complication	intervention directly preceding the incident	medications administered before the incident	strategy	sinu rhythm restored?	admission?
75	K	cardiac arrest	S.C.	propofol 60mg , fentanyl 100 mcg	shock only	no	yes
68	M	TRD	PSA	fentanyl 100 mcg, propofol 150 mg,	shock only	yes	no
66	M	bradycardia	amiodarone	amiodarone 300 mg, antazoline 200 mg, Mg 2g,	meds only	no	no
81	K	bradycardia	antazoline	antazoline 200 mg, Mg 2g, metoprolol 5 mg	meds only	yes	no
66	M	bradycardia	propafenone	antazoline 200 mg, propafenone 105 mg	meds only	no	no

90	K	SVT	antazoline	antaoline 200 mg, amiodarone 300 mg, Mg 2g	meds only	no	no
74	K	SVT	antazoline	antazoline 200 mg, metoprolol 5 mg	meds only	yes	no
63	M	bradycardia	amiodarone	amiodarone 300 mg	combined	yes	no
79	K	bradycardia	propafenone	antazoline 200 mg, propafenone 150 mg	combined	no	yes
45	M	bradycardia	S.C.	propafenone 140mg, Mg 2g, propofol 80 mg, fentanyl 150 mg	combined	yes	yes
75	M	bradycardia	S.C.	amiodarone 450 mg, metoprolol 5 mg, Mg 2g	combined	yes	yes
72	K	chest pain	antazoline	antazoline 200mg, Mg 2g	combined	yes	no
70	M	hypotension	amiodarone	amiodarone 300 mg, Mg 2g	combined	yes	no
49	M	hypotension	antazoline	metoprolol 5 mg, antazoline 200 mg	combined	yes	no
75	K	hypotension	propafenone	antazoline 200 mg, propafenone 70 mg	combined	yes	no
59	M	SVT	antazoline	antazoline 200 mg, Mg 2g	combined	yes	no
65	M	TRD	PSA	amiodarone 300mg, propofol 110mg, fentanyl 100 mcg	combined	yes	no

66	M	TRD	PSA	antazoline 200mg, metoprolol 5 mg	combined	yes	no
65	M	TRD	PSA	antazoline 200 mg, fentanyl 100 mg, propofol 100 mg	combined	yes	no
